# RegEMR: a natural language processing system to automatically identify premature ovarian decline from Chinese electronic medical records

**DOI:** 10.1186/s12911-023-02239-8

**Published:** 2023-07-18

**Authors:** Jie Cai, Shenglin Chen, Siyun Guo, Suidong Wang, Lintong Li, Xiaotong Liu, Keming Zheng, Yudong Liu, Shiling Chen

**Affiliations:** Center for Reproductive Medicine, Department of Gynecology and Obstetrics, Nanfang Hospital, Southern Medical University, Guangzhou, 510515 China

**Keywords:** Diminished ovarian reserve, Electronic medical records, Natural language processing, Ovarian reserve, Premature ovarian failure, Premature ovarian insufficiency

## Abstract

**Background:**

The ovarian reserve is a reservoir for reproductive potential. In clinical practice, early detection and treatment of premature ovarian decline characterized by abnormal ovarian reserve tests is regarded as a critical measure to prevent infertility. However, the relevant data are typically stored in an unstructured format in a hospital’s electronic medical record (EMR) system, and their retrieval requires tedious manual abstraction by domain experts. Computational tools are therefore needed to reduce the workload.

**Methods:**

We presented RegEMR, an artificial intelligence tool composed of a rule-based natural language processing (NLP) extractor and a knowledge-based disease scoring model, to automatize the screening procedure of premature ovarian decline using Chinese reproductive EMRs. We used regular expressions (REs) as a text mining method and explored whether REs automatically synthesized by the genetic programming-based online platform RegexGenerator +  + could be as effective as manually formulated REs. We also investigated how the representativeness of the learning corpus affected the performance of machine-generated REs. Additionally, we translated the clinical diagnostic criteria into a programmable disease diagnostic model for disease scoring and risk stratification. Four hundred outpatient medical records were collected from a Chinese fertility center. Manual review served as the gold standard, and fivefold cross-validation was used for evaluation.

**Results:**

The overall F-score of manually built REs was 0.9444 (95% CI 0.9373 to 0.9515), with no significant difference (paired t test *p* > 0.05) compared with machine-generated REs that could be affected by training set sizes and annotation portions. The extractor performed effectively in automatically tracing the dynamic changes in hormone levels (F-score 0.9518–0.9884) and ultrasonographic measures (F-score 0.9472–0.9822). Applying the extracted information to the proposed diagnostic model, the program obtained an accuracy of 0.98 and a sensitivity of 0.93 in risk screening. For each specific disease, the automatic diagnosis in 76% of patients was consistent with that of the clinical diagnosis, and the kappa coefficient was 0.63.

**Conclusion:**

A Chinese NLP system named RegEMR was developed to automatically identify high risk of early ovarian aging and diagnose related diseases from Chinese reproductive EMRs. We hope that this system can aid EMR-based data collection and clinical decision support in fertility centers.

**Supplementary Information:**

The online version contains supplementary material available at 10.1186/s12911-023-02239-8.

## Background

Infertility/subfertility has affected an estimated 15% of couples across the globe, inflicting great harm on people’s reproductive health along with distress, depression and discrimination [1–3]. It is well established that a low ovarian reserve remains an important cause of infertility [4–6]. Defined as the number of oocytes residing in the ovary, the ovarian reserve reaches its maximum during fetal life and undergoes a slow depletion with the occurrence of ovarian senescence [7]. A growing number of women, however, experience very early aging of the ovaries and become prematurely infertile [8]. The pathological decline in ovarian reserve is a slow progression, and a series of symptoms (e.g., menstrual disturbance) and abnormal-but-not-postmenopausal ovarian reserve test results serve as indicators of different stages, such as diminished ovarian reserve (DOR), premature ovarian insufficiency (POI) and premature ovarian failure (POF) [9, 10]. In clinical practice, using these measurements for early detection of and intervention in premature reproductive decline may help preserve the reproductive potential [11].

Despite their great potential for clinical applications, ovarian reserve data are typically stored in a hospital’s electronic medical record (EMR) system in a narrative text format that is not amenable to data aggregation and analysis. In traditional clinical settings, extracting information from unstructured EMRs requires manual annotation by domain experts, a prohibitively time-consuming and error-prone process insufficient for processing the increasing amount of data. As such, it is desirable to develop automatic methods to parse free text and extract relevant information from clinical narratives in fertility centers.

Natural language processing (NLP) has been reported to enable information retrieval in EMR-based research [12–15]. As a branch of artificial intelligence, NLP is concerned with how to program computers to "understand" human speech and capture useful information from free text such as EMR data. As a fundamental tool for NLP, the rule-based approach relies on identified keywords (such as direction words, location words, and central words) and rule templates to detect a search pattern. The common form of a rule is a regular expression (RE) using a sequence of characters to concisely specify a pattern (e.g., \w + matches a word character) [16, 17]. The rule-based approach yields high accuracy in abstracting numerical values and focused information from lexically constrained data [18–22]. Manually created rules tailored to one specific problem, however, are case-specific, with limited capacity to be generalized to different scenarios. For each particular task, REs should be highly tuned to the intricacies of data, which is time-consuming and costly. To overcome these challenges, machine learning-based methods have been performed to automatically synthesize REs from training text samples [23–26]. The team of Alberto Bartoli reported a genetic programming-based tool to construct REs from scratch [27] and proved that such a tool delivered an F-score comparable to that of humans in many realistic tasks [28]. However, the performance of machine-synthesized REs in extracting information from Chinese EMRs remains unknown.

In this study, we presented a regular expression-based EMR extraction tool (RegEMR) for automated information extraction, detection of premature ovarian decline and classification of related clinical states (DOR, POI and POF) from Chinese reproductive EMRs, with the goal of assisting clinical decision support in reproductive centers.

## Methods

### Datasets

We collected a random sample of 400 outpatient medical records between 2017–9 and 2022–7 from the Center for Reproductive Medicine of Nanfang Hospital. The dataset was randomly divided into training and testing sets. The training set was mainly used for the pattern learning required for RE generation, and the testing set was used to evaluate the performance of the system. We obtained written informed consent from the patients upon admission to our hospital.

### Framework

**Fig. 1:**
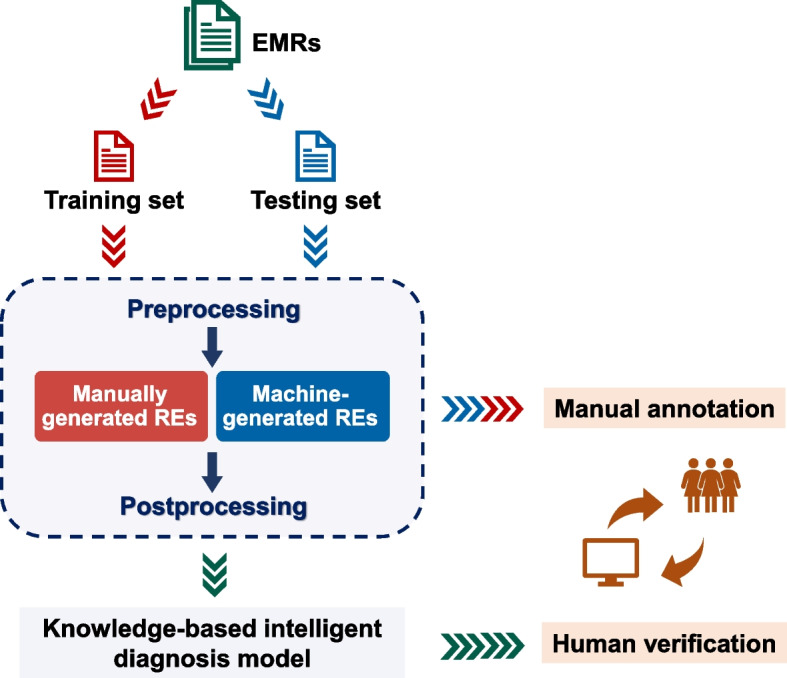
Framework of the study design. EMRs: electronic medical records. RE: regular expression

The whole pipeline of RegEMR is shown in Fig. 1. RegEMR is principally composed of two modules, a rule-based NLP extractor and a knowledge-based disease diagnostic model. The rule-based NLP extractor is capable of extracting relevant information from EMRs through preprocessing, RE conduction, and postprocessing. The extracted information subsequently flows into the diagnostic model for disease scoring and risk stratification, yielding the automated diagnosis of each patient. Given that clinicians typically have insufficient programming skills for RE formulation, we investigate whether machines can substitute for human programmers in RE generation by comparing the effectiveness of manually built REs and machine-synthesized REs in EMR-based extraction tasks. Manual review serves as the gold standard in assessing the performance of automated information extraction and disease detection.

### Rule-based NLP extractor

RegEMR identifies 15 data elements of interest (Table 1), most of which follow an underlying syntactical pattern that can be described by an RE.

**Table 1:** Keywords and rules for concepts of interest

Field	Target concept	Keyword	Rule
Menstrual history	初潮年龄 (Menarche age)	初潮 (Menarche)	C1
	月经周期 (Menstrual cycle)	月经周期 | / (Menstrual cycle)	C1
	月经量 (Menstruation amount)	量 (Amount)	C3
Hormone test	日期 (Date)	年 | 月 | 日 (Year, month, day)	C2
	FSH (Follicle stimulating hormone)	FSH	C1
	LH (Luteinizing hormone)	LH	C1
	E2 (Estrogen)	E2 | E	C1
	P (Progesterone)	P | PRGE	C1
	PRL (Prolactin)	PRL	C1
	T (Testosterone)	T | TEST	C1
	AMH (Anti-Müllerian hormone)	AMH | 抗苗勒管(氏)激素(Anti-Müllerian hormone)	C1
Ultrasonographic measures	子宫内膜厚度 (Endometrial thickness)	内膜(厚) | En (Endometrium)	C1
	子宫位置 (Uterine position)	子宫 | UT (uterus)	C3
	左卵巢卵泡个数 (left antral follicle counting, LAFC)	左(侧)卵巢 | 左(侧)附件 | Lov (Left ovary, left adnexa)	C1
	右卵巢卵泡个数 (right antral follicle counting, RAFC)	右(侧)卵巢 | 右(侧)附件 | Rov (Right ovary, right adnexa)	C1

We developed 3 rule categories. C1: keyword + quantity + unit, e.g., FSH 5.32 mIU/ml; C2: keyword + numeral, e.g., 量中; C3: year + month + day, e.g., 2018年10月3日. Different keywords of the same target concept were split by "|"

#### Preprocessing

Initially, the program preprocessed the deidentified clinical narratives by converting the files to free text as input and removing blank lines. Considering that the relevant data were mostly stored in “现病史”(history of present illness) and “月经史”(menstrual history), we extracted these sections using 2 REs.

#### Manual regular expression generation

When extracting concepts manually from preprocessed data, we empirically determined the keywords indicating that the remainder of the sentence likely contains the target value. For instance, ‘初潮年龄’ (‘Menarche age’) can be indicated by the relevant “hook word” ‘初潮’(‘Menarche’). After locating the labeled sentences, the program applied different rules based on the syntactic and/or semantic co-occurrence patterns found in the training dataset and in domain knowledge (Table 1). Generally, we developed 3 categories of rules: C1 describes a “keyword + quantity + unit” pattern from which a numerical value can be extracted (e.g., hormone level, endometrial thickness, antral follicle counting). A qualitative value such as [‘少’,’中’,’多’…]([‘less’,’medium’,’much’…]) can be obtained utilizing C2, which defines a “keyword + numeral” pattern (e.g., menstruation amount). C3 models the date format. REs were manually compiled in accordance with the developed rules and keywords.

**Fig. 2:**
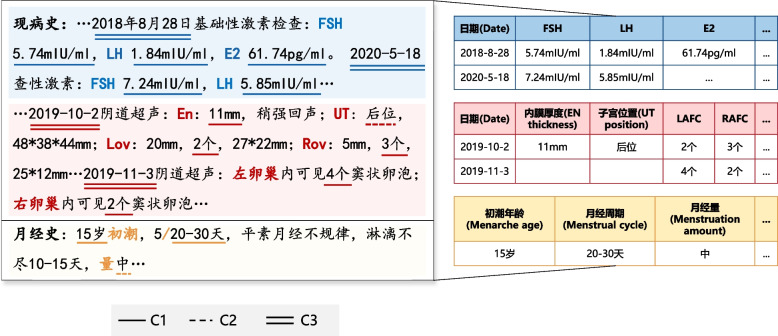
Example of extracting information from a Chinese reproductive clinical record. The color of the font, underlining and table shading are consistent; e.g., a keyword in blue font in the free text has the rule in blue underlining applied to it, yielding the structured table with blue shading

It is common that multiple values exist for one target concept. Some patients might undergo screening tests more than once. Given the necessity to follow up on the dynamic changes in hormone tests and ultrasound measures, we retrieved numerical values and their corresponding dates (date1, measure1; date2, measure2…). We specified a list of the relevant long descriptions labeled by keywords using the previous comma or full stop as a positive lookbehind and the next full stop as a positive lookahead, following the date (Rule C3) and value (Rule C1) searches within each item in the list. For other concepts, the first occurring value is captured. Figure 2 provides an example of extracting the values of predefined concepts from a Chinese reproductive physician’s note.

#### Automated regular expression generation

**Fig. 3:**
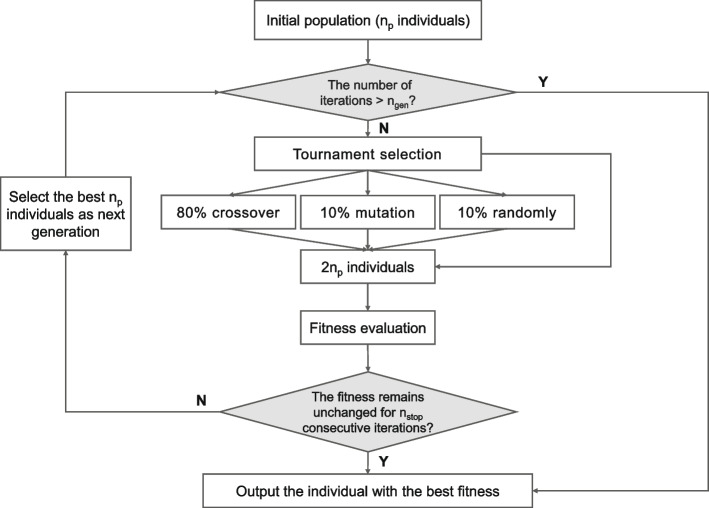
Flowchart of automated regular expression generation using the tree-based genetic programming in [30]

The program obtained machine-generated REs from RegexGenerator +  + (http://regex.inginf.units.it) developed by Bartoli’s team. The platform applies tree-based genetic programming (GP) to evolve regular expressions in several populations (Fig. 3) [29, 30]. Each RE (individual) is encoded as a syntax tree with a depth-first post-order visit. The node labels of the tree are composed of a set of predefined basic constructs of REs (e.g., \d, \w, a-z, A-Z,0–9, = ,\?, +  + ,? + ,* +) and some sequences of characters with high occurrence (> 90%) in the desired extractions of the learning datasets as “building blocks” (e.g., ‘FSH’ could be a useful “building block” to extract the FSH level). The construction of the initial population (${n}_{p}$ individuals) is partly random and partly based on the analysis of the learning dataset; i.e., for each example, 4 different REs are built to ensure the extraction of the corresponding desired portions, which provides a satisfactory starting point for successive evolution. The quality of the candidate solutions is evaluated using a multiobjective optimization algorithm with two fitness indexes: the extraction performance (to be maximized) and the length of REs (to be minimized). The evolution of individuals is performed in an iterative process following an elitism strategy. At each iteration, ${n}_{p}$ new individuals are generated: 10% at random with a ramped half-and-half method, 10% by mutation of the current population, and 80% by crossover of the current population (a tournament size of 7 is used for the selection of individuals undergoing genetic operators). Subsequently, the program ranks the resulting 2 ${n}_{p}$ individuals based on their fitness. The best ${n}_{p}$ individuals are selected as members of the next generation, which are typically on average more fit than the previous ones. Iteration terminates when a predefined maximum generation number (${n}_{gen}$) is reached or a solution with perfect fitness is obtained (i.e., the fitness is fixed for ${n}_{stop}$ consecutive iterations). The string transformation of the best individual at the last iteration is the output of GP execution. It is noteworthy that the algorithm includes ‘or’ operator ‘|’ when a single RE exhibits perfect precision (1.00) yet is not capable of expressing multiple patterns or formats. Such a separate-and-conquer strategy contributes to an appropriate trade-off between specificity and generality.

We imported snippets from the training dataset and annotated the desired substrings to be extracted as examples to train the model. Based on these examples, the system generated REs. Since the automated construction of REs is based solely on user-provided examples, its effectiveness depends critically on the degree to which the examples represent the extraction task. In this study, we characterized the representativeness of annotated examples from the perspectives of quantity and quality. A larger learning corpus may span an increasing diversity and density of expression styles to better cover the semantic features of the desired extractions. Therefore, we varied the training set size (80%, 40% or 20% of the full dataset) for each task to investigate how the number of examples affects the performance of the generated REs. In addition, focusing on snippets containing the keywords and lexical structures that are commonly seen may be more important than overfocusing on scarce and “difficult” examples that bring no significant improvements in overall performance. Therefore, we assessed the performance of the automated extractor with different degrees of emphasis on keywords. We annotated both keywords and values and compared the results to those obtained when only values were annotated. In the former case, if a keyword has a high occurrence in annotations, it can be identified as a useful “building block” for the construction of the initial population.

#### Postprocessing

A stepwise postprocessing workflow was developed to identify and discard outliers in the extracted data, including checking for numeric values outside specific ranges, detecting typos entered by clinicians, removing duplicate values within one EMR, and identifying possible errors based on the category of the value (e.g., dates instead of values).

### Evaluation

To evaluate the performance of the rule-based extractor, we had two medical students perform manual abstraction. A third student was in charge of addressing inconsistencies between the two annotations. Then, the manual annotation served as the gold standard to which the rule-based extractions were compared. The pair-wise inter-annotator agreement results are shown in Supplementary Table 1 (Table S1). True positive (TP) means the extracted value matched that in the reference; false positive (FP) means the extracted value did not match that in the reference; true negative (TN) means no value was extracted and there was no reference value; false negative (FN) means no value was extracted but there was a reference value. We calculated the three standard metrics of precision, recall and F-score following the formulas below. Five-fold cross-validation was conducted and these three standard metrics were averaged across the five repetitions.

1 $$\begin{array}{c}Precision=\frac{TP}{TP+FP}\end{array}$$

2 $$\begin{array}{c}Recall \left(Sensitivity\right)=\frac{TP}{TP+FN}\end{array}$$

3 $$\begin{array}{c}F-score=\frac{2\times Precision\times Recall}{Precision+Recall}\end{array}$$

### Knowledge-based diagnostic model

In clinical practice, abnormal ovarian reserve testing serves as a fundamental indicator of fertility dysfunction. Specifically, elevated basal serum FSH (follicle-stimulating hormone) is a specific, but not sensitive, test for diminished ovarian reserve [7]. AMH (anti-Mullerian hormone) levels and, alternatively, AFC (the sum of antral follicle counts in both ovaries) are more sensitive to a subtle decline in ovarian reserve [7]. The existence of distinct yet related diseases, specifically DOR, POI and POF commonly complicate the diagnostic and research scenario given that their definition and diagnosis are based on ovarian reserve [8]:

- DOR: FSH > 10 IU/L, AMH ≤ 1 ng/mL, AFC ≤ 5 [8, 10, 31].
- POI: amenorrhoea or menstrual irregularity, FSH > 25 IU/L [6, 32].
- POF: amenorrhoea or menstrual irregularity, FSH > 40 IU/L [10].

(FSH and AFC are required to be tested on two occasions > 4 weeks apart.)

There are no absolutely clear boundaries among these three diseases, and they can be regarded as a continuum of ovarian conditions: the “occult” clinical state (reduced fecundity but normal FSH levels and regular menses), the “biochemical” state (reduced fecundity, elevated FSH and regular periods, closely corresponding to DOR), and the “overt” state (reduced fecundity, elevated FSH and irregular periods, approximately corresponding to POI or POF) [33].

**Fig. 4:**
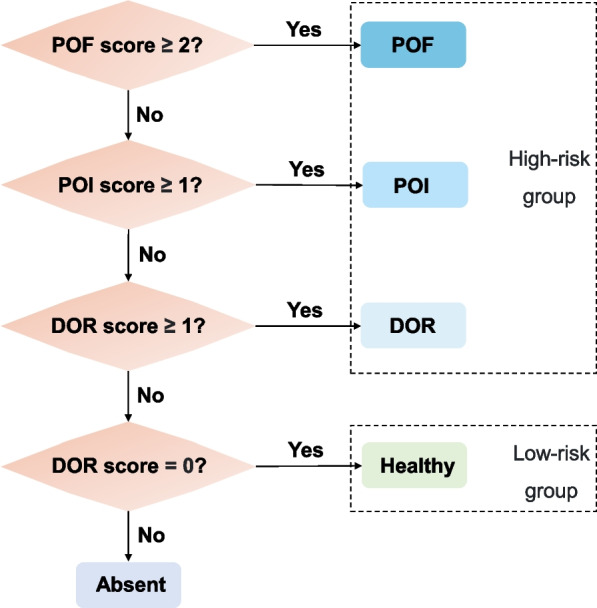
Flowchart for the automatic diagnosis and risk stratification model. The DOR, POI, and POF scores were calculated as in Table 2. We classified the machine diagnosis of “DOR”, “POI” and “POF” as the high-risk group, and “Healthy” as the low-risk group

To identify people with declining ovarian reserve and evaluate which clinical state (DOR, POI or POF) they have in an automated manner, we proposed a scoring model to annotate, translate and encode the clinical diagnostic criteria (Table 2). Since ‘irregular menses’ does not have a standard definition, we quantified it using a < 24- or > 35-day cycle. The score was a null value if a related description could not be detected (AFC was null when left or right AFC was null). The sum of each subsection score was the total score, representing the risk of each clinical state. The POI and POF scores were calculated only when the subject was younger than 40; otherwise, they were given a null value. If the diagnostic criteria were strictly obeyed, only full marks indicating that all criteria were met could be used to make a diagnosis of a specific state. However, the information recorded in EMRs might be incomplete, and completing the medical history could require multiple encounters. In this case, the occurrence of even one abnormal but pivotal result could reveal potential risk. Therefore, we loosened the inclusion criteria and constructed a risk stratification model as described in Fig. 4. We classified the diagnosis of “DOR”, “POI” and “POF” as the high-risk group requiring early intervention, and “Healthy” as the low-risk group.

**Table 2:** The scoring model of DOR, POI and POF

	Criteria	Score
**The scoring model of DOR**		
AFC ≤ 5	Absent data	Null
	On no occasion	0
	Only on one occasion	1
	On at least two occasions < 4 weeks apart	2
	On at least two occasions > 4 weeks apart	3
AMH	Absent data	Null
	> 1 ng/mL	0
	≤ 1 ng/mL	3
FSH > 10 IU/L	Absent data	Null
	On no occasion	0
	Only on one occasion	1
	On at least two occasions < 4 weeks apart	2
	On at least two occasions > 4 weeks apart	3
Sum		DOR Score
**The scoring model of POI**		
Menstrual cycle	Absent data	Null
	24–35 days	0
	< 24 or > 35 days	1
FSH > 25 IU/L	Absent data	Null
	On no occasion	0
	Only on one occasion	1
	On at least two occasions < 4 weeks apart	2
	On at least two occasions > 4 weeks apart	3
Sum		POI Score
**The scoring model of POF**		
Menstrual cycle	Absent data	Null
	24–35 days	0
	< 24 or > 35 days	1
FSH > 40 IU/L	Absent data	Null
	On no occasion	0
	Only on one occasion	1
	On at least two occasions < 4 weeks apart	2
	On at least two occasions > 4 weeks apart	3
Sum		POF Score

The total score is the sum of the scores of its subsections. The POI and POF scores are calculated only for women under the age of 40

We validated the model using NLP extractions combined with age in the structured EMR data. The program conducted data preprocessing by unit conversion, numeric format unification, and converted the date into a numeric format to calculate the interval between examinations. The minimum value was used for calculation when the AMH levels tested on several occasions were all documented. Clinical diagnosis by specialized technicians was used as the gold standard to assess the performance of our model.

## Results

We counted the absent values for each concept in the manual annotation of the training and testing datasets (Table S2). The proportion of uncharacterized data varied for different concepts. The mean and variance of each numerical variable were also calculated (Table S3). The results showed that features pertaining to ovarian reserve presented high interpatient variability, indicating their clinical and prognostic value.

**Fig. 5:**
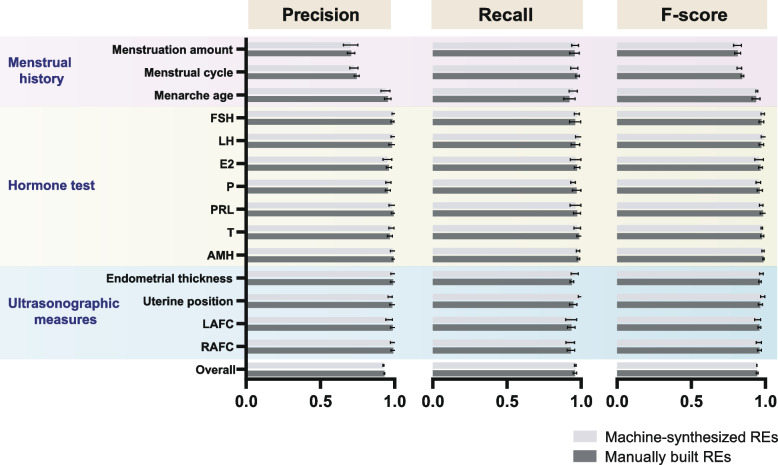
The performance of each target concept using manually created versus machine-generated regular expressions (REs)

The rule-based extractor demonstrated great performance using both manually formulated and machine-generated REs. Figure 5 and Table S4 summarize the average performance indexes (precision, recall and F-score) over five repetitions and their 95% confidence intervals. Overall, manually built RE extraction in the local test set was 93.13% (95% CI 92.83% to 93.43%) specific and 95.79% (95% CI 94.58% to 97.00%) sensitive, and the REs produced by machine performed with a comparable precision of 0.9253 (95% CI 0.9210 to 0.9296) and recall of 0.9601 (95% CI 0.9547 to 0.9656). To compare the results obtained by these two approaches, we conducted a paired t test (the difference in performance indexes followed a normal distribution). The results exhibited no significant difference in precision, recall and F-score (*p* > 0.05, *p* = 0.079, 0.648, 0.644, respectively), demonstrating the ability of machines to compete with human operators in RE generation. A practically perfect agreement between the rule-oriented extractor and manual abstraction, with an average F-score greater than 0.94, was observed in hormone tests and ultrasonographic measures, indicating that our NLP extractor was capable of tracing the dynamic changes in these measurements. However, a lower accuracy was found in 'Menstrual cycle' and 'Menstruation amount' for both manually constructed (0.7449 and 0.7073) and machine-synthesized REs (0.7255 and 0.7042). With a limited capacity to track the progression of menstruation described in a lexically free format, the current rule-based extractor might only extract part of the menstruation information when multiple values exist, which principally accounts for the low precision.

**Fig. 6:**
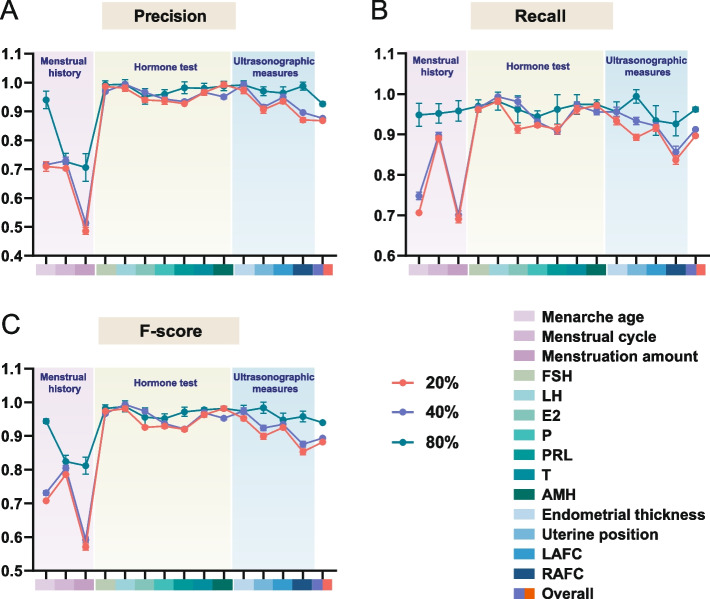
The performance of each target concept with different training set sizes

**Fig. 7:**
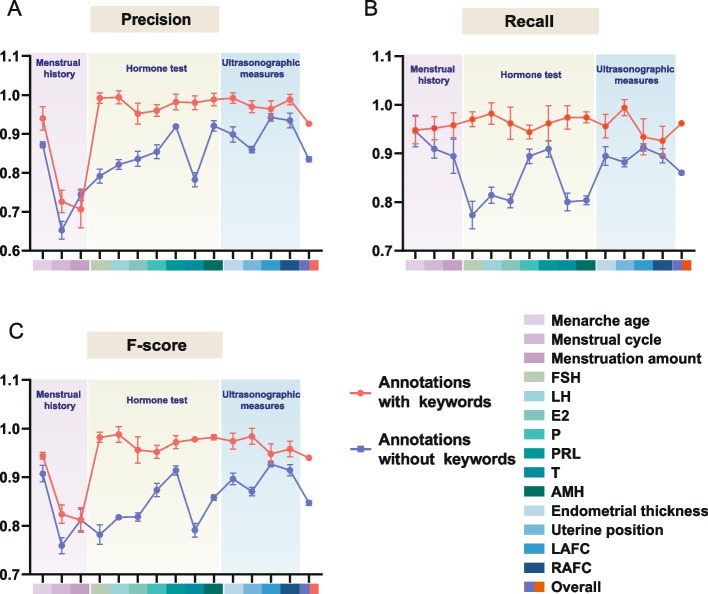
The performance of each target concept with different annotation portions

To gain insight into how the representativeness of examples is related to the effectiveness of the GP-based extractor, we repeated our experiments with different training set sizes (Fig. 6) and different annotation portions (Fig. 7) and plotted the average performance indexes in the testing set. As shown in Fig. 6, the performance of the generated REs differed significantly for training set ratios of 80%, 40% and 20% (Friedman test for F-score, *p* = 2.8e-5 < 0.001). Generally, a higher ratio for learning was accompanied by a better performance, which was particularly obvious in ‘Menstrual history’ when the ratio rose from 40 to 80%. In contrast, such improvement was less apparent for ‘Hormone test’ and ‘Ultrasonic measures’ with an increasing number of provided examples. For the extraction of ‘Hormone test’ and ‘Ultrasonic measures’, a relatively small percentage of data was always enough to represent the general pattern, which was relatively fixed; thus, an acceptable performance could be obtained even when the learning information included only 20% or 40% snippets. The description of ‘Menstrual history’, however, was highly varying, with semantic constraints that a few examples could not describe. In this scenario, the 20% or 40% learning corpus was too small for the machine to perform as effectively as with more learning material. Increasing the number of available training examples remarkably improved performance. Similarly, the paired t test for F-score exhibited a significant difference (*p* = 1.02e-4 < 0.01) between annotations with and without keywords. Annotations with keywords typically yielded higher effectiveness, ‘Hormone test’ in particular. The description of ‘Hormone test’ contained the levels of different hormones, which closely resembled each other because they were all numerical values. Annotating only the values might not be adequately representative of the data, confusing the extractor and yielding irrelevant numbers. For instance, for the extraction of FSH levels, the annotation ‘FSH 12.53 IU/L’ gave more priority to the keyword 'FSH' than ‘12.53 IU/L’, which might be more important for machines to infer a general pattern from the example.

**Fig. 8:**
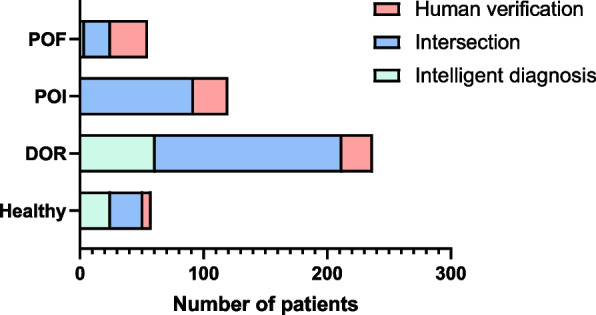
Comparison of machine diagnosis and human verification for 380 patients

We validated the knowledge-based diagnostic model and calculated the disease score on the full dataset (*n* = 400). The data of 20 subjects were insufficient for machine diagnosis and thus were eliminated. The distribution and confusion matrix for disease diagnosis and risk stratification are shown in Fig. 8 and Table S5, respectively. The model demonstrated desirable effectiveness in risk screening, with high accuracy (0.9803) and sensitivity (0.9330). For each specific disease, the automated diagnosis in 76% of patients (*n* = 290) was consistent with that of the clinical diagnosis, and the kappa coefficient was 0.63. Otherwise, the statistical analysis of the score echoed the rules of disease progression (Table 3), indicating the plausibility and clinical interpretability of the model.

**Table 3:** Descriptive statistical analysis of machine diagnosis

Machine diagnosis	DOR score	POI score	POF score
Healthy	0	0	0
DOR	3.68 ± 1.76	0	0
POI	4.37 ± 1.66	1.13 ± 0.34	0.85 ± 0.36
POF	4.72 ± 1.72	3.04 ± 0.61	3.04 ± 0.61

## Discussion

In the last decade, neural network models, including long short-term memory (LSTM), conditional random field (CRF), and bidirectional encoder representations from transformers (BERT), have significantly advanced the field of medical text mining. For instance, ASUDS applied a bidirectional LSTM (BiLSTM) model to detect pediatric substance use information from clinical notes [34]. Med7 used a named-entity recognition model for the identification of drug-related information [35]. Leiter et al. captured symptoms of congestive heart failure by adopting a GraphIE model that used a graphical structure to abstract relations between words [36, 37]. Liu et al. proposed a pretrained, fine-tuned BERT-based BiLSTM-CRF model to recognize evidence for liver cancer diagnosis [38]. BioBERT [39] and ClinicalBERT [40] are domain-specific BERT-based models pretrained on biomedical data. Indeed, these deep learning methods have boosted the development of biomedical NLP. Nonetheless, the large amounts of medical data required for training are commonly not available for Chinese hospitals or departments. At present, Chinese clinical data from many institutions are available only internally because of considerations of patient privacy and information security. Additionally, neural networks are based on a “black-box” theory, making it difficult to interpret the parameters and improve the results. In this regard, REs are commonly used because of their simplicity, flexibility and interpretability. REs can be designed faster, and can potentially be applied to a variety of clinical settings, which is especially important when commercial NLP tools are unaffordable to researchers.

To automatically extract information from EMRs, many English medical language processing tools have been developed, such as MedLEE [41], cTAKES [42], MetaMap [43] and MedTagger [44]. However, these tools cannot be directly transplanted to Chinese EMR tasks due to the differences between the Chinese and Western languages. In contrast to the burgeoning NLP toolkits for English, few studies have focused on Chinese NLP systems. Several existing Chinese NLP tools, such as HanLP (hanlp.linrunsoft.com/index.html), ICTCLAS [45], and Fudan NLP [46], are not specifically designed for EMR-based extraction tasks. To the best of our knowledge, RegEMR is the first Chinese text analysis pipeline applied to the field of reproductive medicine. The rule-based NLP extractor module used manually created versus machine-generated REs to transform textual clinical information into structured, analyzable data from Chinese reproductive EMRs. It performed well in tracking dynamic changes in hormone and ultrasonographic measures with high accuracy and recall rates comparable to those of human abstractors. In this regard, this work might provide a good solution for data collection and aggregation in fertility centers.

We validated the performance of machine-synthesized REs in extracting information from Chinese EMRs and demonstrated that they performed competitively with human-formulated REs. Table S6 lists some examples of word patterns that were correctly and incorrectly interpreted by current extraction systems. Most of the false negatives were due to other unexpected word patterns not captured by the set of REs used; typically, these patterns occurred rarely. Many of the false positive identifications were attributed to less specific rules triggering noise. A common goal for both human operators and machine learning was to establish a balance between generalization and overfitting. The programmers generalized patterns principally based on domain knowledge and effectively formulated keyword-centered REs that were readable but could easily lead to overfitting. The automated system inferred the actual desired behavior from the examples and synthesized REs that were able to address inexact keyword matching but could be difficult to interpret. If the effectiveness of the obtained solutions is adequate for practical usage, the automatic RE generator may prove to have good performance in clinical applications because it requires no specific skills or familiarity with the syntax of REs, and has appreciable scalability and transferability to other diseases and departments. With this in mind, although the datasets of this study were from the same hospital, we believe that the automatic RE generator module could be transferred to EMRs of other centers that use different formats. In the present study, we also revealed that the representativeness of the examples might affect the performance of automated RE extractor. Autogeneration of high-quality REs demanded sufficient learning corpora and annotations in representativeness of the text, while the specific requirements varied across different tasks. Training with representative examples enables improved performance.

Recent years have witnessed multiple studies focused on applying artificial intelligence (AI) to reproductive medicine, including automation of follicle counts and prediction of embryo cell stages [47]. However, the automated identification of declining ovarian reserve has not been achieved. To automatize the diagnostic procedure, we translated the clinical diagnostic criteria into a programmable disease scoring model that took into account the characteristics of reproductive EMRs and real clinical work. Our knowledge-based diagnostic model demonstrated acceptable performance with high sensitivity and specificity in risk screening and a 0.63 kappa coefficient compared with manual diagnosis. This scoring model can also be directly transplanted to EMRs of other languages as a prescreening tool to aid in the diagnosis of DOR, POI and POF.

This study still has several limitations. First, the sample size of the study is limited. A relatively consistent expression style of EMRs from one center might lead to overfitting by algorithms. A multicenter dataset may help enhance the robustness and adaptability of RegEMR. Second, machine-synthesized REs are based solely on examples, and users must dig into the large set of input text to identify snippets whose annotations may indeed be useful. In the future, we will try to overcome this challenge by including an interactive learning procedure: the user marks only one desired extraction and then merely answers extraction queries generated by the system [48]. Third, the menstruation information extracted by the current rule-based method is incomplete when menstruation fluctuation occurs, which might interfere with the accuracy of machine diagnosis produced by the knowledge-based scoring model. Enhancing the precision and recall of NLP methods might help bolster this system. Fourth, machine diagnosis is solely based on the values documented in EMRs and cannot distinguish whether these values are pre- or posttherapy. Taking more relevant information (e.g., past medical history) into consideration might help improve the performance of the automatic diagnosis model. Finally, RegEMR is mainly an algorithmic process. User-friendly interfaces are required for the further application and promotion of this tool.

## Conclusion

Clinical detection of DOR, POI, and POF relies on manual recognition of multiple measurements comprising the diagnostic criteria that are typically documented in EMRs but are not synthesized. We propose RegEMR, an artificial intelligence tool composed of a rule-oriented NLP extractor and a knowledge-based disease scoring model, to automatize the diagnostic procedure using Chinese reproductive EMRs. The NLP extractor of RegEMR is the first Chinese NLP system specifically designed for information extraction from reproductive EMRs. It presents high accuracy (manual RE: 0.9313, machine RE: 0.9253) and recall rates (manual RE: 0.9579, machine RE: 0.9601) comparable to those of human abstractors, with desirable performance in automatically tracing dynamic changes in hormone levels (F-score 0.9518–0.9884) and ultrasonographic measures (F-score 0.9472–0.9822). Additionally, we annotated and translated the clinical diagnostic criteria into a programmable disease scoring model, which demonstrated acceptable performance with high sensitivity (0.93) and specificity (0.98) in risk screening and a 0.63 kappa coefficient compared with manual diagnosis. Our work provides possibilities for automatic screening and diagnosis of premature reproductive decline from EMRs. Our follow-up work will continue to increase the datasets to verify and refine the algorithms and develop user-friendly interfaces for their real-world application. We hope that RegEMR will reduce the workload of EMR-based information extraction and aid in risk detection and disease diagnosis in fertility centers.

## Supplementary Information

**Additional file 1.**

## Data Availability

The datasets used and/or analyzed during the current study are available from the corresponding author on reasonable request.

## Abbreviations

NLP: Natural language processing
EMR: Electronic medical record
RE: Regular expression
DOR: Diminished ovarian reserve
POI: Premature ovarian insufficiency
POF: Premature ovarian failure
AFC: Antral follicle count
FSH: Follicle stimulating hormone
LH: Luteinizing hormone
E2: Estradiol
P: Progesterone
PRL: Prolactin
T: Testosterone
AMH: Anti-Müllerian hormone
AFC: Antral follicle count
