# Supplementary material for: RegEMR: a natural language processing system to automatically identify premature ovarian decline from Chinese electronic medical records
Source: BMC Med Inform Decis Mak. 2023 Jul 18;23:126. doi: 10.1186/s12911-023-02239-8 (PMC10353087; doi:10.1186/s12911-023-02239-8)
Supplement: Supplementary file 1 — Additional file 1. [file 12911_2023_2239_MOESM1_ESM.docx]

**Table S1** Pair-wise inter-annotator agreement results between each annotator and the gold standard

| Field | Target concept | Annotator-1 | Annotator-2 |
| --- | --- | --- | --- |
| Menstrual history | Menarche age | 1.00 | 0.99 |
|  | Menstrual cycle | 0.92 | 0.95 |
|  | Menstruation amount | 1.00 | 0.98 |
| Hormone test | FSH | 1.00 | 1.00 |
|  | LH | 1.00 | 1.00 |
|  | E2 | 1.00 | 0.97 |
|  | P | 0.95 | 1.00 |
|  | PRL | 1.00 | 0.98 |
|  | T | 0.96 | 0.99 |
|  | AMH | 0.96 | 1.00 |
| Ultrasonographic measures | Endometrial thickness | 0.98 | 0.95 |
|  | Uterine position | 0.94 | 0.96 |
|  | LAFC | 0.99 | 1.00 |
|  | RAFC | 1.00 | 0.96 |
| Overall |  | 0.98 | 0.98 |

#### **Table S2** The number of absent values and its proportion in the data set

| Field | Target concept | Total absent ratio |
| --- | --- | --- |
|  |  |  |
| Menstrual history | Age | 0.00 |
|  | Menarche age | 0.40 |
|  | Menstrual cycle | 0.08 |
|  | Menstruation amount | 0.18 |
| Hormone test | FSH | 0.25 |
|  | LH | 0.26 |
|  | E2 | 0.29 |
|  | P | 0.33 |
|  | PRL | 0.57 |
|  | T | 0.44 |
|  | AMH | 0.21 |
| Ultrasonographic measures | Endometrial thickness | 0.51 |
|  | Uterine position | 0.50 |
|  | LAFC | 0.40 |
|  | RAFC | 0.43 |

**Table S3** Clinical characteristics of patients

|  | Mean ± SD |
| --- | --- |
| Age (years) | 34.66±4.76 |
| Menarche age (years) | 13.46±1.59 |
| Menstrual cycle(days) | 28.88±9.54 |
| FSH (IU/L) | 41.85±37.10 |
| LH (IU/L) | 21.21±23.94 |
| E2 (pg/mL) | 39.71±56.27 |
| P (ng/mL) | 0.82±2.70 |
| PRL (ng/mL) | 25.17±47.15 |
| T (ng/mL) | 2.45±9.21 |
| AMH (ng/mL) | 0.48±0.71 |
| Endometrial thickness(mm) | 6.77±3.69 |
| LAFC | 2.84±2.92 |
| RAFC | 2.84±3.11 |

#### **Table S4** The average precision, recall and F-score of each target concept across five repetitions

|  | Target concept | Precision | Recall | F-score |
| --- | --- | --- | --- | --- |
| Menstrual history | Menarche age | 0.9544  (0.9384) | 0.9204  (0.9464) | 0.9369  (0.9419) |
|  | Menstrual cycle | 0.7449  (0.7255) | 0.9751  (0.9536) | 0.8445  (0.8238) |
|  | Menstruation amount | 0.7073  (0.7042) | 0.9553  (0.9592) | 0.8125  (0.8114) |
| Hormone test | FSH | 0.9864  (0.9935) | 0.9598  (0.9708) | 0.9726  (0.9820) |
|  | LH | 0.9840  (0.9516) | 0.9611  (0.9833) | 0.9721  (0.9884) |
|  | E2 | 0.9618  (0.9516) | 0.9721  (0.9623) | 0.9669  (0.9568) |
|  | P | 0.9544  (0.9591) | 0.9696  (0.9447) | 0.9618  (0.9518) |
|  | PRL | 0.9944  (0.9820) | 0.9724  (0.9616) | 0.9832  (0.9713) |
|  | T | 0.9684  (0.9785) | 0.9865  (0.9732) | 0.9773  (0.9756) |
|  | AMH | 0.9938  (0.9877) | 0.9812  (0.9778) | 0.9874  (0.9826) |
| Ultrasonographic measures | Endometrial thickness | 0.9899  (0.9902) | 0.9379  (0.9562) | 0.9631  (0.9728) |
|  | Uterine position | 0.9851  (0.9698) | 0.9469  (0.9949) | 0.9655  (0.9822) |
|  | LAFC | 0.9875  (0.9628) | 0.9335  (0.9326) | 0.9595  (0.9472) |
|  | RAFC | 0.9911  (0.9869) | 0.9307  (0.9260) | 0.9598  (0.9553) |
| Overall |  | 0.9313  (0.9253) | 0.9579  (0.9601) | 0.9444  (0.9424) |

Values out of the parentheses are the results for human-generated regular expressions. Values in the parentheses are the results for machine-generated regular expressions.

#### **Table S5** Confusion matrix for disease diagnosis and risk classification applied to testing dataset

|  | Reference standard | | | |
| --- | --- | --- | --- | --- |
|  | Healthy | DOR | POI | POF |
| Machine diagnosis |  | | | |
| Healthy | 26 | 25 | 0 | 0 |
| DOR | 7 | 151 | 24 | 30 |
| POI | 0 | 0 | 92 | 0 |
| POF | 0 | 0 | 4 | 21 |

|  | Reference standard | |
| --- | --- | --- |
|  | Low-risk group | High-risk group |
| Machine classification |  | |
| Low-risk group | 26 | 25 |
| High-risk group | 7 | 322 |

#### **Table S6** Examples of true positive (TP), false positive (FP) and false negative (FN) in NLP extractor

| Target concept | TP | FP | FN |
| --- | --- | --- | --- |
| Menarche age | - 13岁初潮^a^ (Menarche at the age of 13) - 初潮：13岁^a^ (Menarche: 13) | - 平素月经规律，4-5/25天，量中。^b^ (Regular menstruation, 4-5/25 days, moderate.) - 初潮17岁，平素月经规律，7/35天，量中。^b^ (Age 17 at menarche, regular menstruation, 7/35 days, moderate.) | 月经史：13，量少。^a^ (Menstrual history: 13, small amount.) |
| Menstrual cycle | - 7/28-30天^a^ (7/28-30 days) - 10/2-3个月^a^ (10/2-3 month) | - 芬吗通2/10mg ^a^ (Fenmaton 2/10mg) - 2019/09/09 ^a^ - 月经史：初潮14岁，月经周期规律，7/26-27天。2022年1月开始出现月经周期延长至35天。^a^ (Menstrual history: 14 years old at menarche, regular menstrual cycle, 7/26-27 days. In January 2022, the menstrual cycle will be extended to 35 days) | 月经周期约28天^b^ (The menstrual cycle is approximately 28 days) |
| Menstruation amount | - 量少^a^ (Hypomenorrhea) - 量适中^a^ (Moderate menstruation) - 量偏多^a^ (Excessive menstruation) - 量较少^a^ (Fewer menstruation) - 量正常^a^ (Normal menstruation) | - 骨量正常^a^ (Normal bone mass) - 月经史：初潮14岁，量中。2022年1月开始月经量较前减少1/3。^a^ (Menstrual history: 14 years old at menarche, moderate amount. In January 2022, the amount of menstruation will decrease by 1/3 compared with the previous period. ) | - 月经量较前少^c^ (The amount of menstruation is less than before) - 量少4年。^b^ (Hypomenorrhea for 4 years) |
| Hormone test | - 性激素检查：FSH 21.93mIU/ml，LH8.5 mIU/ml，E2：35.36pg/ml，P0.99 ng/ml，PRL 15.23ng/ml，T30.76 ng/dl ^a^ (Sex hormone test: FSH 21.93mIU/ml, LH8.5 mIU/ml, E2: 35.36pg/ml, P0.99 ng/ml, PRL 15.23ng/ml, T30.76 ng/dl) - FSH100+IU/L ^a^ - E2<5 pg/ml ^a^ - TEST 0.320ng/mL ^a^ - PRGE 0.132ng/ml ^a^ | E2-80.78pg/ml ^a^ | - 2017-10-14查基础性激素：FSH 8.47，LH3.02。^c^ (On October 14, 2017, the basic sex hormones test: FSH 8.47, LH3.02) - FSH波动在8.92-26.95mIU/ml ^c^ (FSH fluctuates between 8.92-26.95mIU/ml) - 患者自诉有多次性激素结果，FSH均大于25 ^c^ (The patient complained of multiple sexual hormone results, with FSH greater than 25) - prl 3.54ng/ml ^b^ - E46.55pg/ml ^b^ - 抗苗勒管激素：5.310ng/mL ^b^ (Anti-Müllerian hormone: 5.310ng/mL) |
| Endometrial thickness | En：6mm ^a^ |  | - Em：8mm ^c^ - 内膜厚3.8mm ^b^ (Endometrial thickness is 3.8 mm) |
| Uterine position | - UT：前位^a^ (UT: anterior position) - 子宫前位^a^ (Anterior position of uterus) | UT 43*34*44mm ^b^ | UT平位^c^ (UT horizontal position) |
| AFC | LOV：3-4个，28*16mm，ROV：2-3个，实性低回声团31*14mm，39*20mm ^a^ (LOV: 3-4, 28 * 16mm, ROV: 2-3, solid hypoechoic group: 31 * 14mm, 39 * 20mm. ) | 左11-12个^b^ (left: 11-12) | - Lov：未见AFC，13*6mm。^b^ (Lov: no antral follicles could be found, 13 * 6mm.) - 双侧卵巢未见AFC。^a^ (No antral follicles could be found in bilateral ovaries) - LOV：32*19mm，内见一无回声暗区，22*17*20mm。^a^ (LOV: 32 * 19mm, a dark area without echo can be seen inside, 22 * 17 * 20mm) - 双侧卵巢窦状卵泡均3个^a^ (There were 3 antral follicles on both sides of the ovary. ) |

#### ^a^: in both NLP extractors; ^b^: only in the automated NLP extractor; ^c^: only in the manual NLP extractor.
